# Supplementary figures and images for: Population genomic and evolutionary modelling analyses reveal a single major QTL for ivermectin drug resistance in the pathogenic nematode, Haemonchus contortus
Source: BMC Genomics. 2019 Mar 15;20:218. doi: 10.1186/s12864-019-5592-6 (PMC6420744; doi:10.1186/s12864-019-5592-6)

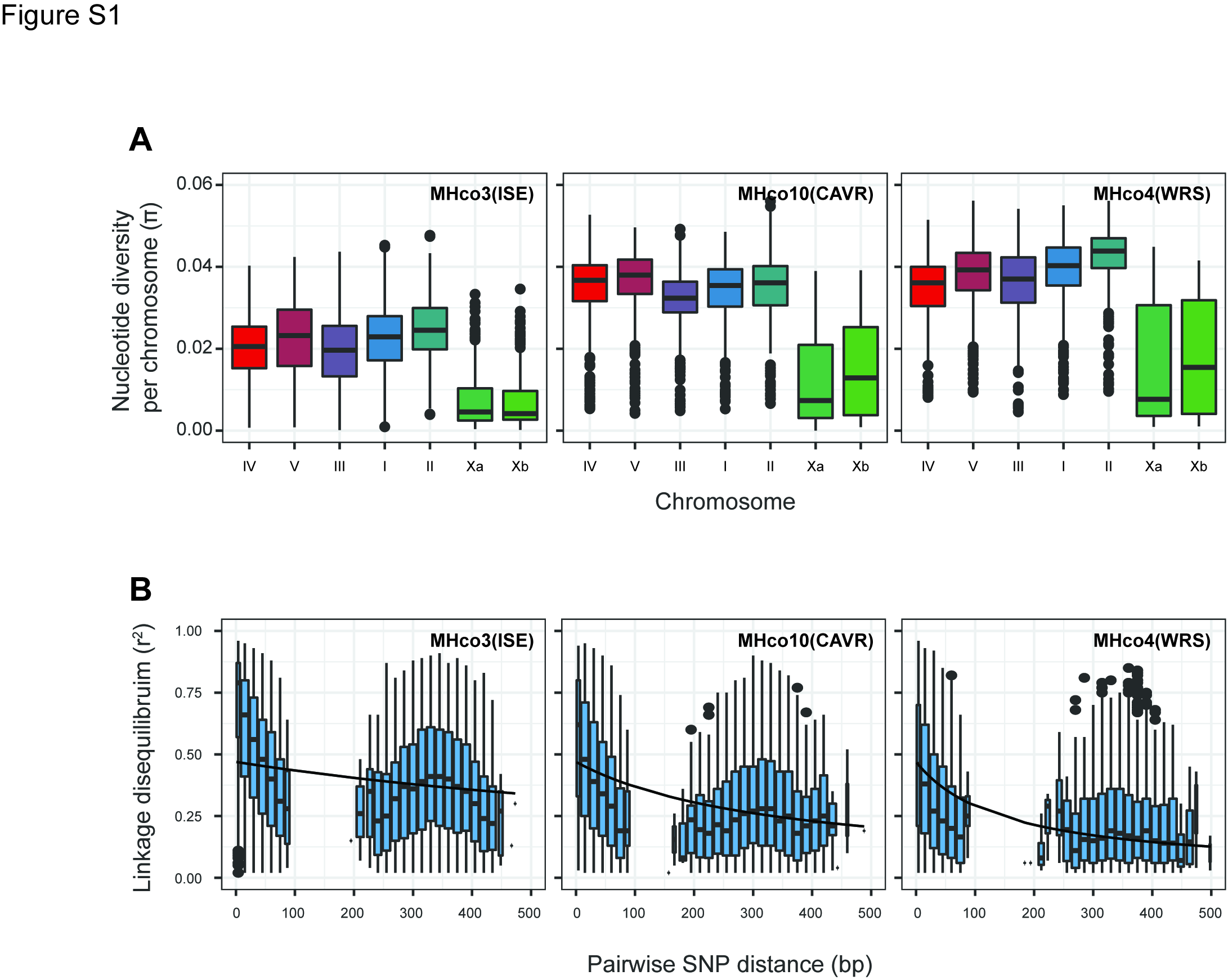

Supplement: Supplementary file 1 — Figure S1. Characterisation of within-population diversity. A. Within population nucleotide diversity per chromosome, summarising genome-wide data presented in Fig. 2c. Colours represent chromosomes as described in Fig. 2a. B. Linkage disequilibrium between variants present in paired reads was estimated using LDx for each parental population. Line represents expected LD decay over genetic distance [60, 61]. (TIF 16681 kb) [file 12864_2019_5592_MOESM1_ESM.tif]

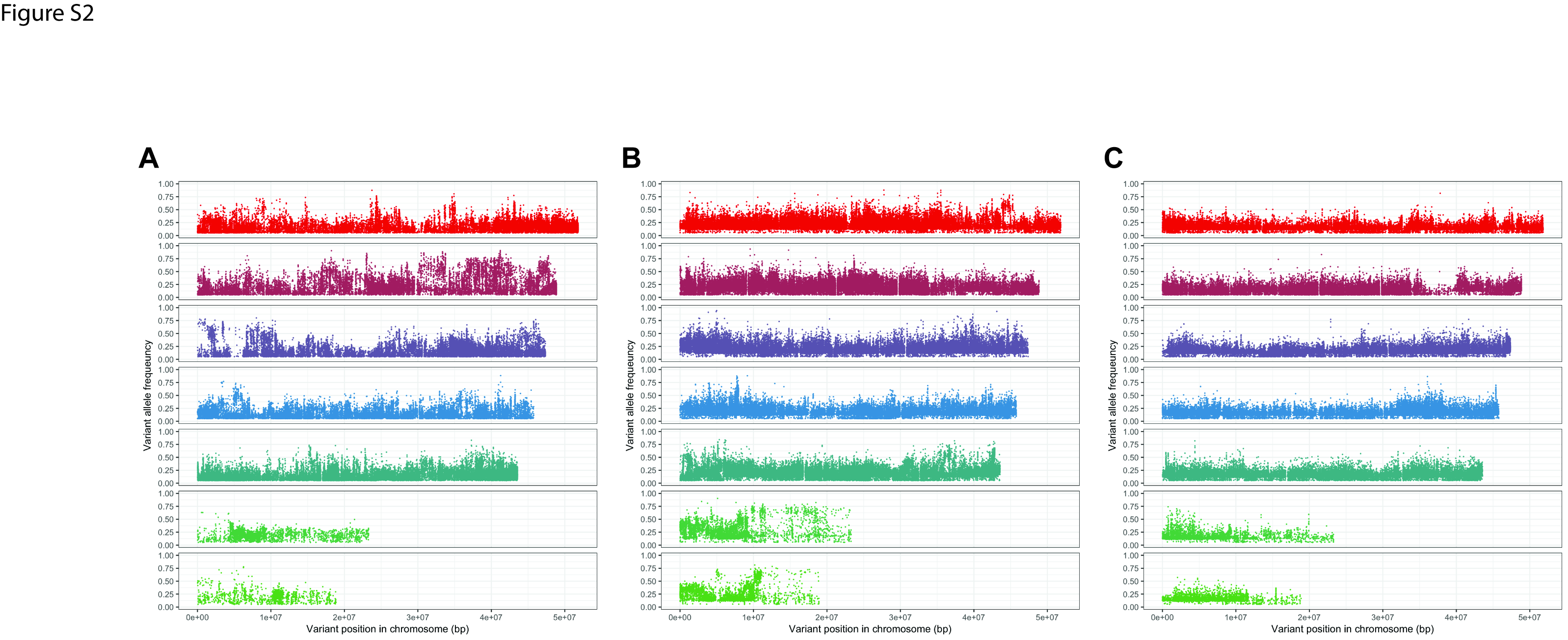

Supplement: Supplementary file 2 — Figure S2. Distribution of “private” variant sites per parental population. A. MHco3(ISE). B. MHco10(CAVR). C. MHco4(WRS). Private sites were defined as having a frequency greater than 0.05 in the population of interest, but less than 0.05 in the two additional populations. (TIF 19122 kb) [file 12864_2019_5592_MOESM2_ESM.tif]

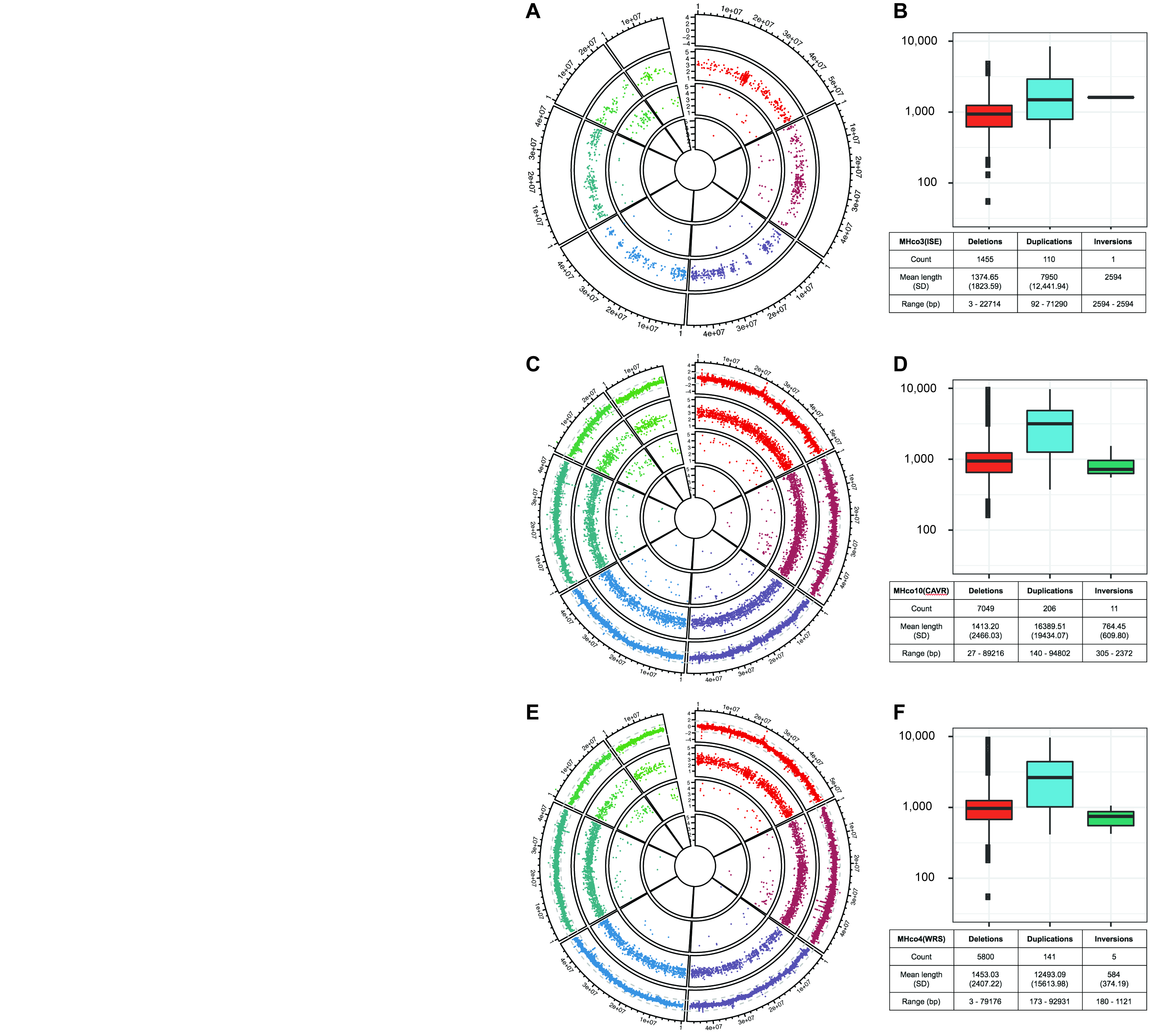

Supplement: Supplementary file 3 — Figure S3. Copy number and structural variation in the parental lines. A,B. MHco3(ISE), C,D. MHco10(CAVR), E,F. MHco4(WRS). Data per circos plot (A,C,E) is orientated as follows; outer circle: CNV variation between MHco3(ISE) and each resistant parent. No CNV comparison was made in A; second circle: deletions; third circle: duplications; inner circle: inversions. The data presented in A,C,E is summarised in the boxplots (feature length distribution) and tables in B,D,F. (TIF 40104 kb) [file 12864_2019_5592_MOESM3_ESM.tif]

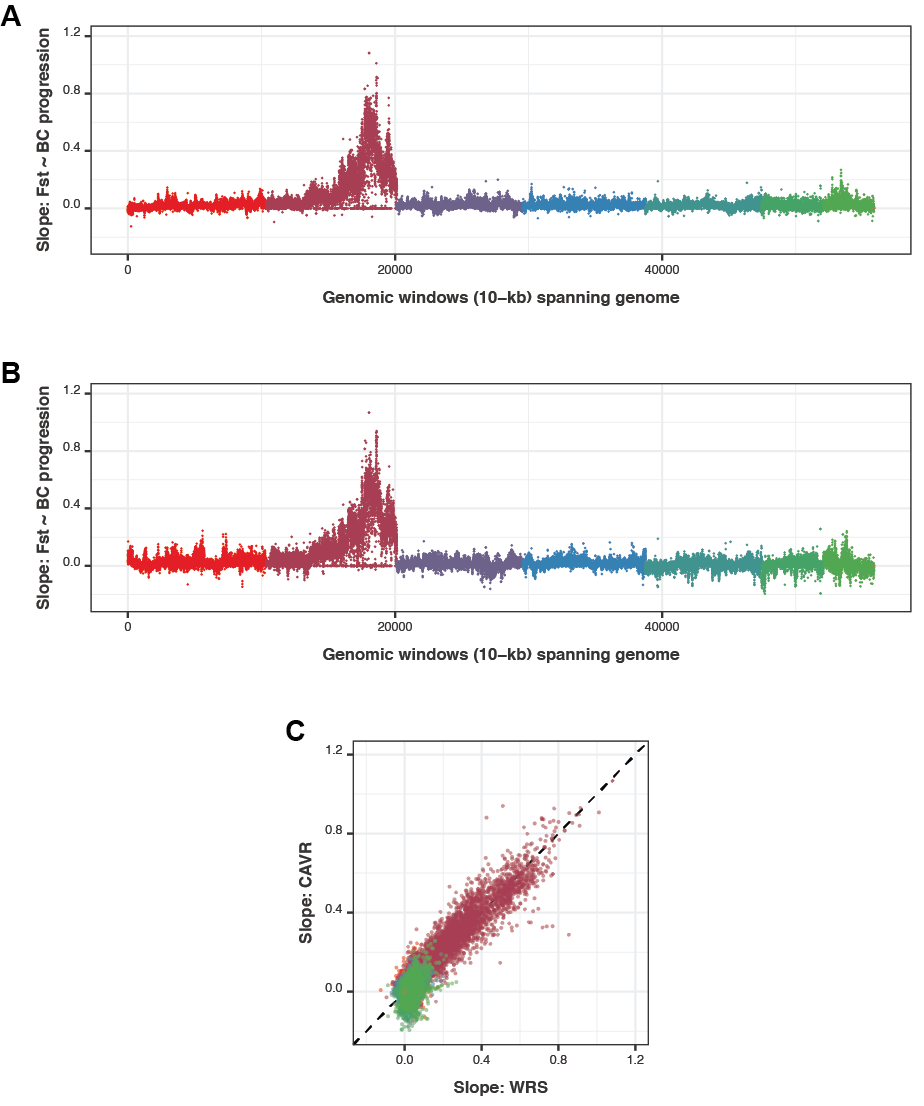

Supplement: Supplementary file 4 — Figure S4. Summary of genome-wide change in FST throughout the backcross and subsequent passage. Linear regression between FST and the four sampling time points was performed for each 10 kbp window sampled across the genome for both MHco3/10 (A) and MHco3/4 (B). The slope of the regression was plotted. Panel C shows the correlation between the slopes (FST vs backcross progression) for MHco3/10 (A) and MHco3/4 (B). The dashed line represents x = y. Colours represent chromosomes as described in Fig. 2a. (PNG 140 kb) [file 12864_2019_5592_MOESM4_ESM.png]

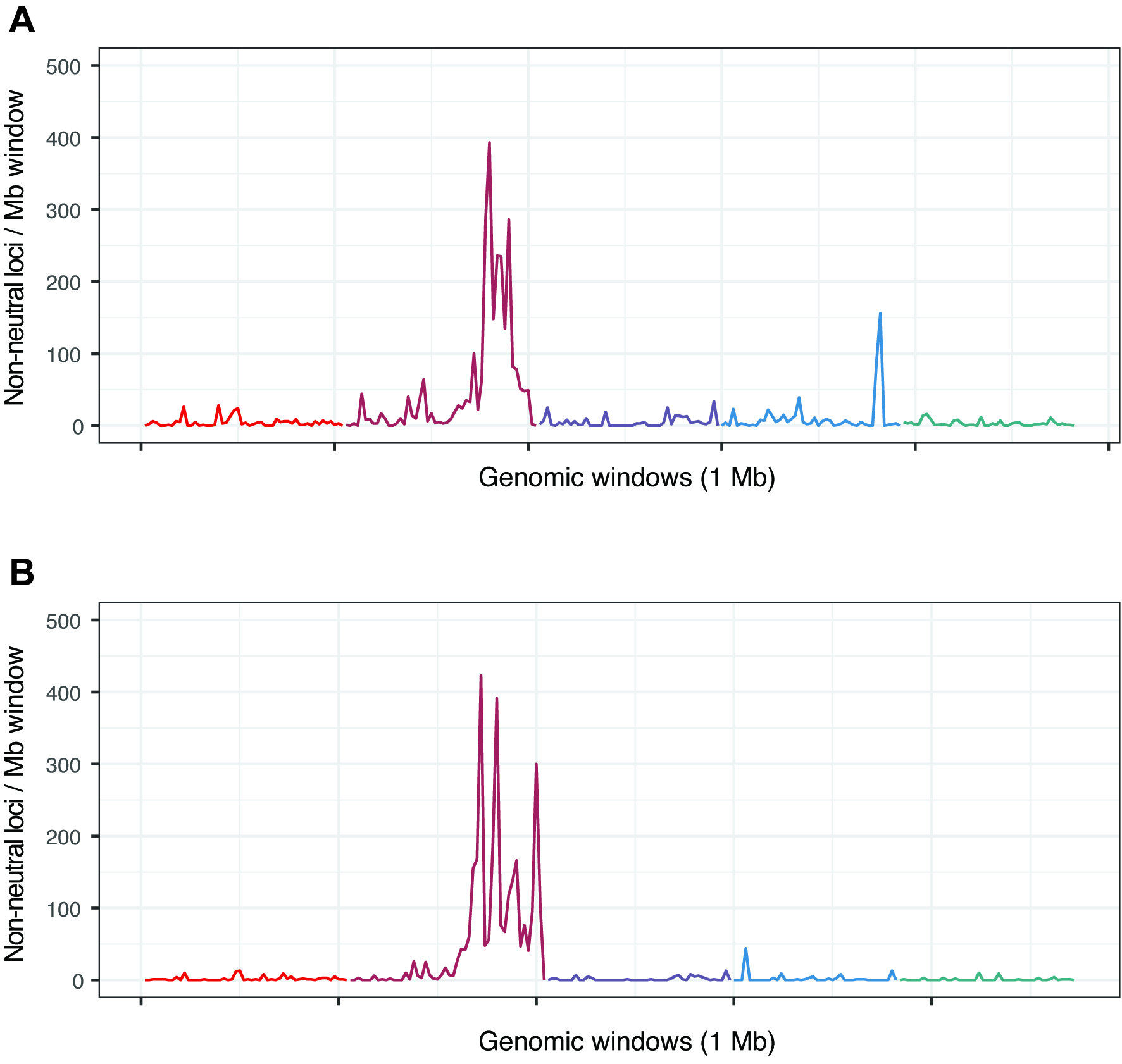

Supplement: Supplementary file 5 — Figure S6. Location of significantly non-neutral loci identified using the single-locus population genetic model. Corresponding peaks in the location of significant sites can be seen in the MHco3/10 (A) and MHco3/4 datasets (B). A total of 70.6% of significant sites in the MHco3/10 dataset, and 90.6% of significant sites in the MHco3/4 dataset, were found in chromosome V. Data are binned in 1 Mbp windows spanning the genome. Colours represent chromosomes as described in Fig. 2a. (TIF 13064 kb) [file 12864_2019_5592_MOESM5_ESM.tif]

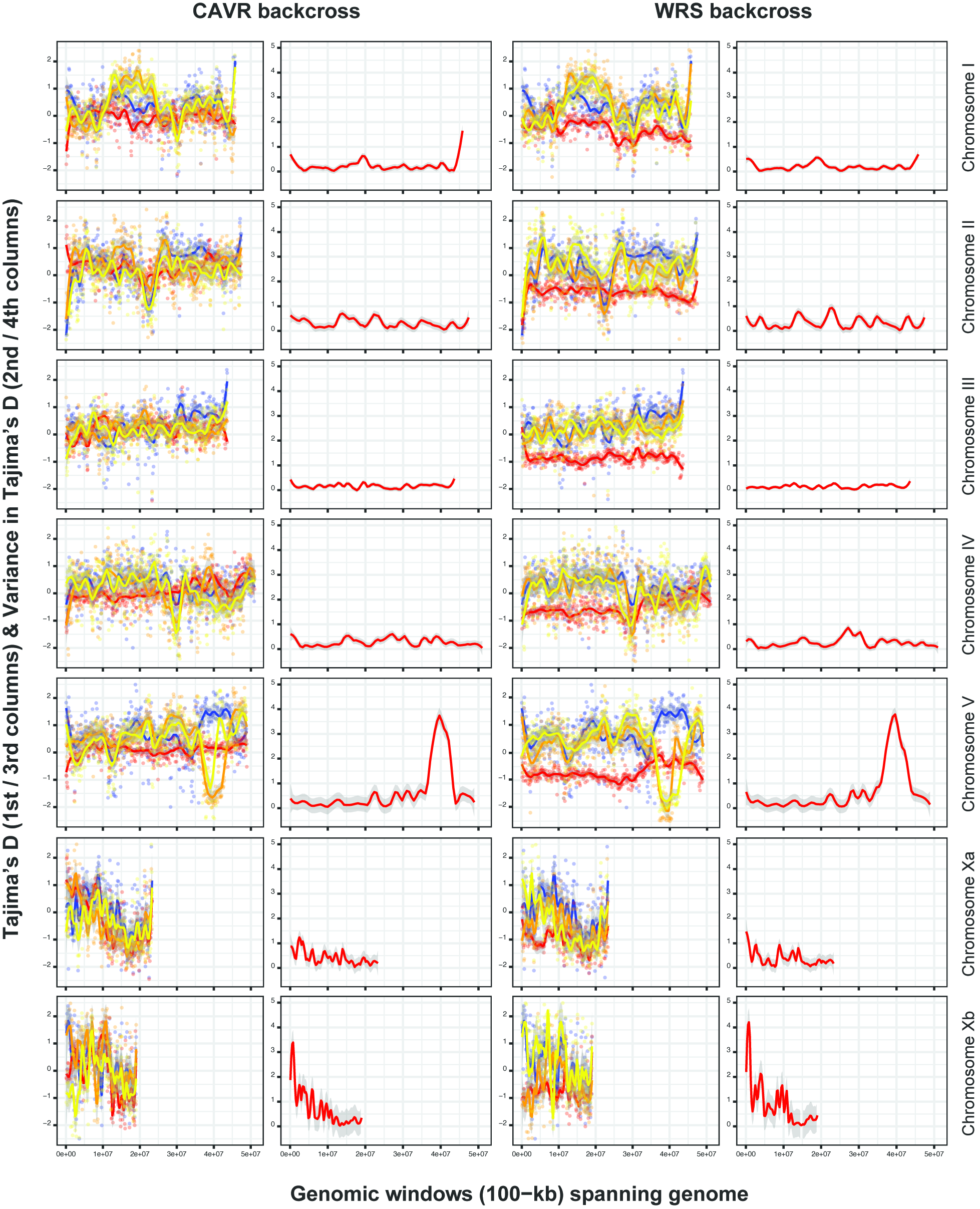

Supplement: Supplementary file 6 — Figure S7. Analysis of Tajima’s D variation in each chromosome per cross. Comparison of Tajima’s D per chromosome between MHco3(ISE) parent (blue), MHco10(CAVR) (panel column 1; red) or MHco4(WRS) (panel column 3; red) and passages 3 (orange) and 4 (yellow) of the crosses. Tajima’s D was calculated using npstat in 100 kbp windows spanning the genome. The variance in the mean value of Tajima’s D among MHco3(ISE) and passages 3 and 4 – for which an increase in variance would suggest introgression and evidence of selection – was determined and is presented as smoothed line (red) in panel columns 2 and 4. (TIF 23273 kb) [file 12864_2019_5592_MOESM6_ESM.tif]

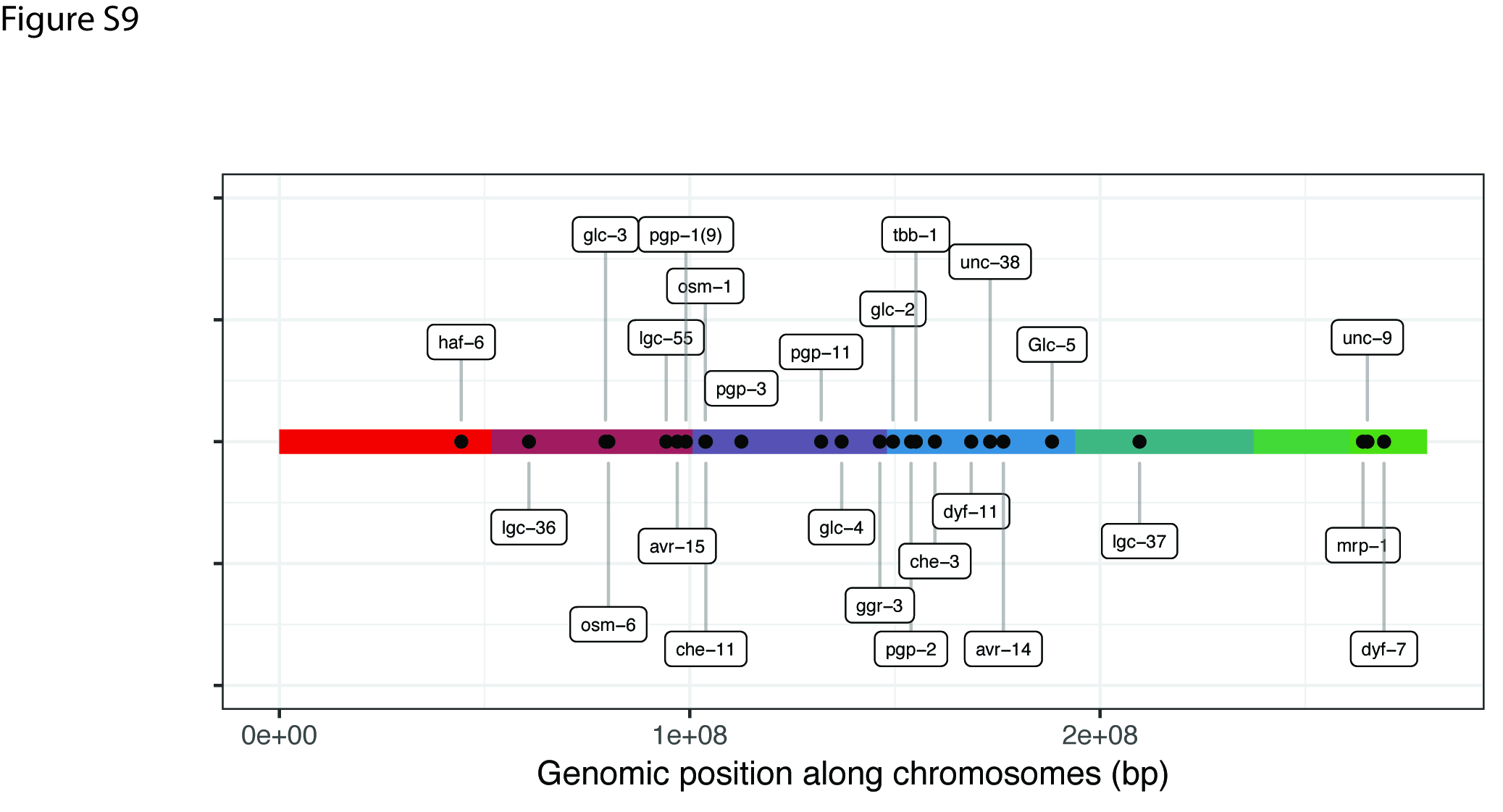

Supplement: Supplementary file 8 — Figure S9. Relative position and location of candidate genes from the literature proposed to be associated with ivermectin resistance in Haemonchus contortus and/or Caenorhabditis elegans. Gene coordinates are presented in S2 Table. Colours represent chromosomes as described in Fig. 2a. (TIF 10253 kb) [file 12864_2019_5592_MOESM8_ESM.tif]

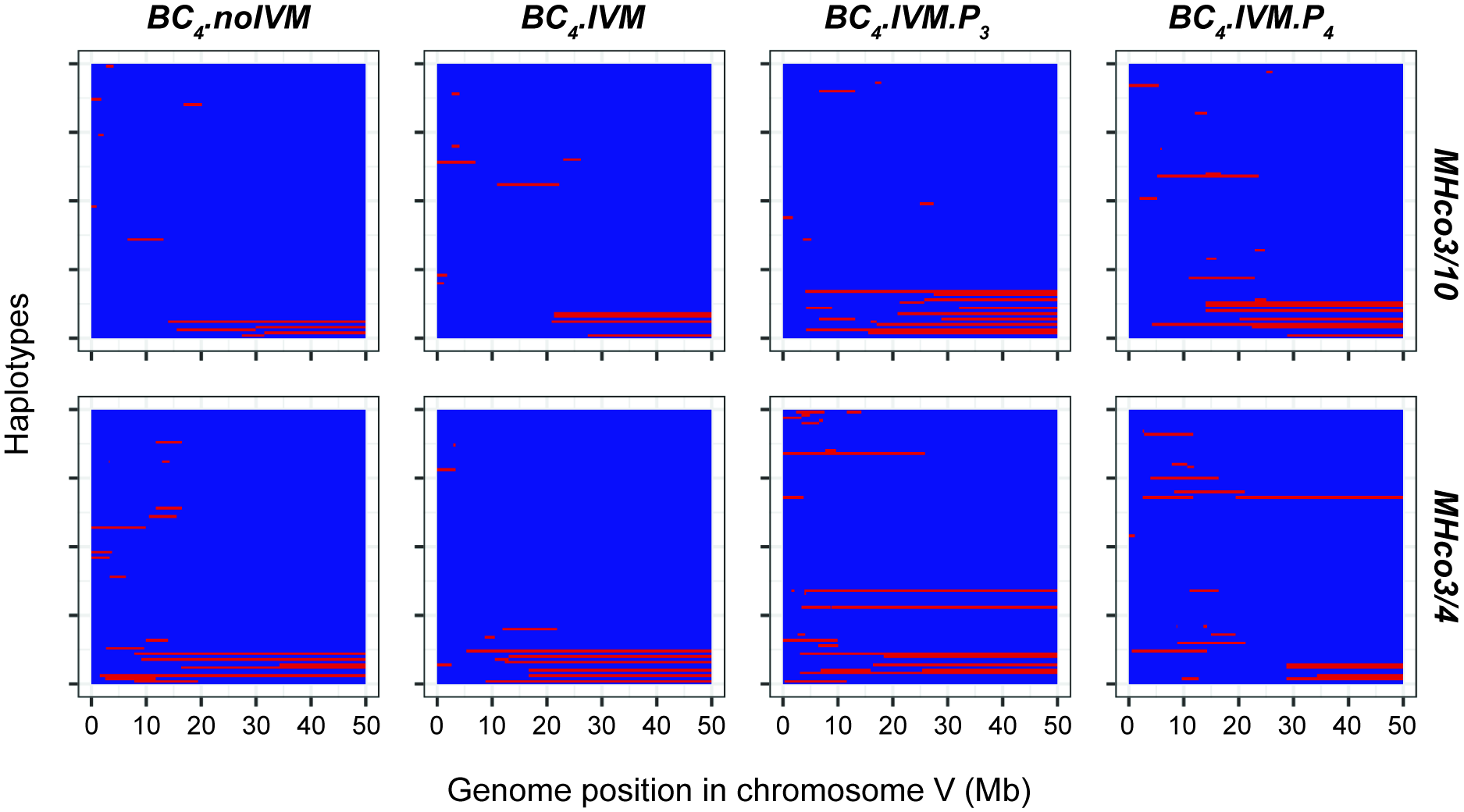

Supplement: Supplementary file 9 — Figure S10. Haplotype structure of chromosome V in an example output from the model under neutral evolution. Segments of genome from the resistant parent are shown in red, while segments of genome from the susceptible parent are shown in blue. The repeated backcross removes most of the resistant genotypes from the population. (TIF 8743 kb) [file 12864_2019_5592_MOESM9_ESM.tif]

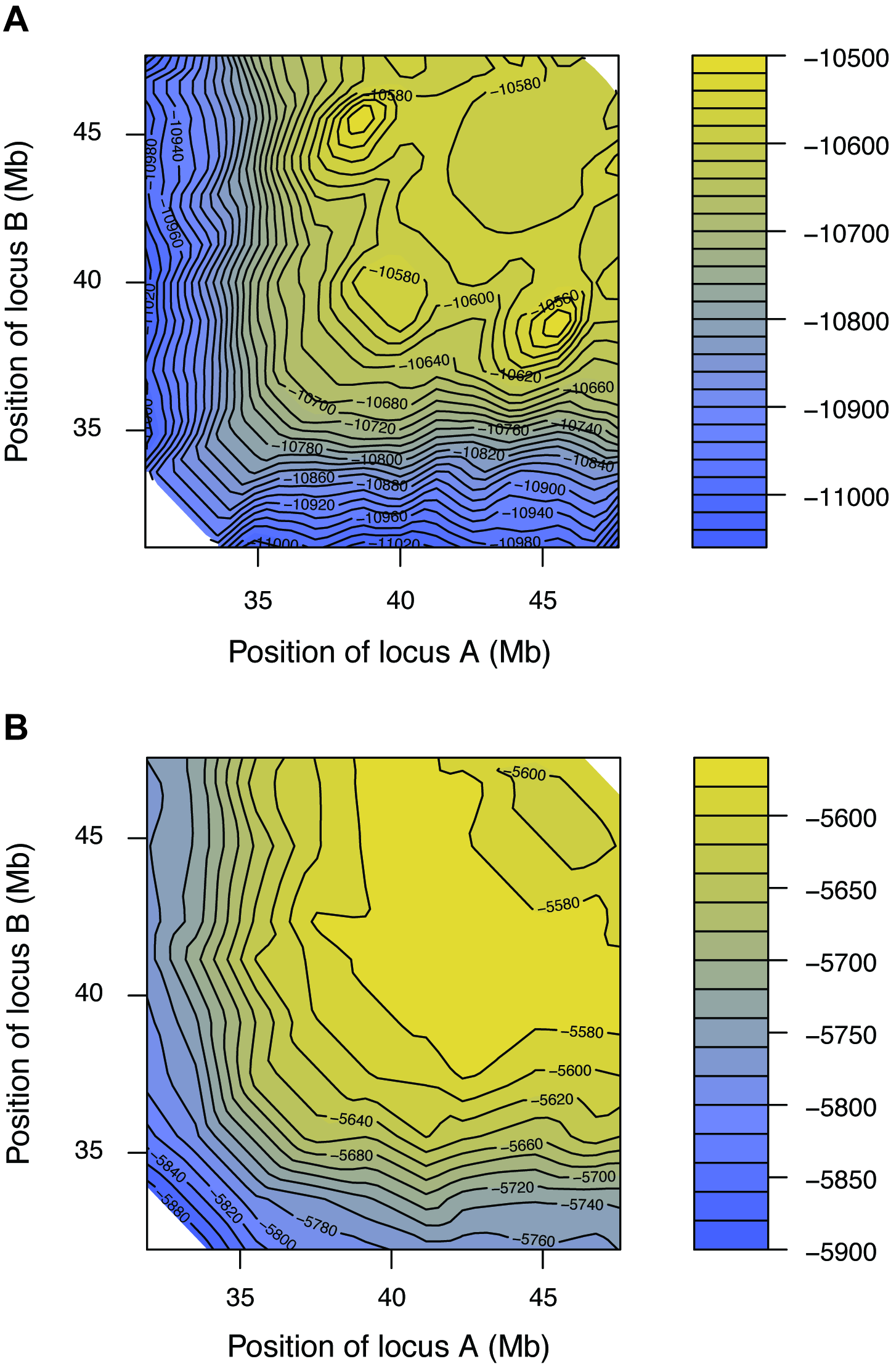

Supplement: Supplementary file 10 — Figure S8. Contour maps of log likelihood scores derived from the two locus driver model. A. MHco10(CAVR). B. MHco4(WRS). The model was restricted to interactions between pairs of loci at least 2 Mbp apart. (TIF 14861 kb) [file 12864_2019_5592_MOESM10_ESM.tif]

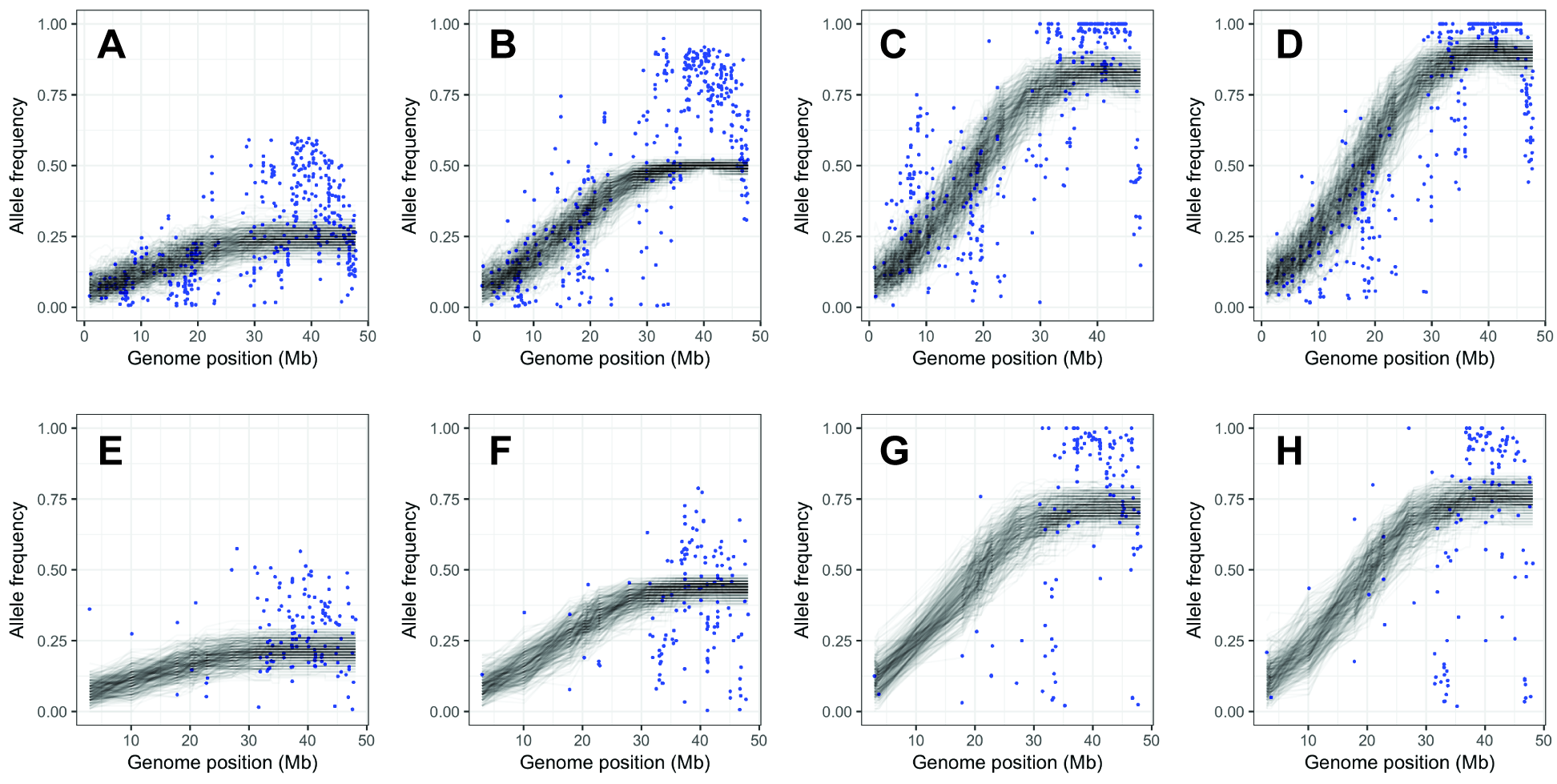

Supplement: Supplementary file 11 — Figure S5. Fits between the model and the data for each data sample. Blue dots show filtered allele frequencies for putative segregating sites. The model fit is shown as gray lines; a distinct line is shown for each of the 250 replicate simulations run for the parameters generating the maximum likelihood fit. A. MHco3/10.BC4.noIVM. B. MHco3/10.BC4.IVM. C. MHco3/10.BC4.IVM.P3. D. MHco3/10.BC4.IVM.P4. E. MHco3/4.BC4.noIVM. F. MHco3/4.BC4.IVM. G. MHco3/4.BC4.IVM.P3. H. MHco3/4.BC4.IVM.P4. (TIF 9801 kb) [file 12864_2019_5592_MOESM11_ESM.tif]
