# Supplementary material for: Population genomic and evolutionary modelling analyses reveal a single major QTL for ivermectin drug resistance in the pathogenic nematode, Haemonchus contortus
Source: BMC Genomics. 2019 Mar 15;20:218. doi: 10.1186/s12864-019-5592-6 (PMC6420744; doi:10.1186/s12864-019-5592-6)
Supplement: Supplementary file 7 — Table S2. Candidate genes from literature proposed to be associated with ivermectin resistance in Haemonchus contortus and/or Caenorhabditis elegans. (DOCX 18 kb) [file 12864_2019_5592_MOESM7_ESM.docx]

**S2 Table. Candidate genes from literature proposed to be associated with ivermectin resistance in *Haemonchus contortus* and / or *Caenorhabditis elegans***

| **Gene** | **GeneID ^3^** | **Chromosome** | **Coordinates ^4^** | **Reference** |
| --- | --- | --- | --- | --- |
| avr-14b / gbr-2 or HcGluClα3 *^2^* | HCOI00274000, HCOI00274100 | I | 28352540 - 28362260 | [(Williamson et al., 2011)](https://paperpile.com/c/DAmyjC/WDbr) |
| avr-15 | HCOI00130400 | V | 45145104 - 45158307 | [(Dent et al., 2000)](https://paperpile.com/c/DAmyjC/Ip09) |
| tbb-1 | [HCOI01967600](https://parasite.wormbase.org/Haemonchus_contortus_prjeb506/Gene/Summary?g=HCOI01967600) | I | 7026384 - 7029311 | [(Eng et al., 2006; de Lourdes Mottier and Prichard, 2008)](https://paperpile.com/c/DAmyjC/nYUr+wYZZ) |
| che-3 ^1^ | [HCOI01710300](https://parasite.wormbase.org/Haemonchus_contortus_prjeb506/Gene/Summary?g=HCOI01710300) | I | 11666953 - 11695321 | [(Dent et al., 2000)](https://paperpile.com/c/DAmyjC/Ip09) |
| dyf-11 ^1^ | HCOI01514900 | I | 20494101 - 20505946 | [(Dent et al., 2000)](https://paperpile.com/c/DAmyjC/Ip09) |
| dyf-7 | HCOI01065900 | Xb | 8461163 - 8465904 | [(Urdaneta-Marquez et al., 2014)](https://paperpile.com/c/DAmyjC/5uOx) |
| ggr-3 | [HCOI00098800](https://parasite.wormbase.org/Haemonchus_contortus_prjeb506/Gene/Summary?g=HCOI00098800) | II | 45626226 - 45639348 | [(Rao et al., 2009)](https://paperpile.com/c/DAmyjC/fqi4) |
| glc-1 ^1^ | NA |  |  | [(Dent et al., 2000)](https://paperpile.com/c/DAmyjC/Ip09) |
| glc-2 / GluClb ^2^ | HCOI00383000 | I | 1443439 - 1446121 |  |
| glc-3 | HCOI00543300 | V | 27643249 - 27655188 | [(Williamson et al., 2011)](https://paperpile.com/c/DAmyjC/WDbr) |
| glc-4 | [HPLM_0001206301](https://parasite.wormbase.org/Haemonchus_placei_prjeb509/Gene/Summary?g=HPLM_0001206301) | II | 36314509 - 36331683 |  |
| glc-5 / HcGluCla ^2^ | HCOI00617300 | I | 40196936 - 40206359 | [(Blackhall et al., 1998)](https://paperpile.com/c/DAmyjC/Omx7) |
| lgc-36 | HCOI02054500 | V | 9008092 - 9028181 |  |
| lgc-37 / HG-1 ^2^ | HCOI00977100 | III | 15741515 - 15762899 | [(Blackhall et al., 2003)](https://paperpile.com/c/DAmyjC/5b70) |
| lgc-55 | HCOI00162900 | V | 42450788 - 42469177 |  |
| osm-1 ^1^ | HCOI00970500 | II | 3105809 - 3126440 |  |
| osm-3 | HCOI00164700, HCOI00673700 |  |  |  |
| osm-5 ^1^ | HCOI00498400, HCOI00077900 |  |  |  |
| pgp-1 / pgp-9 ^2^ | HCOI00233200 | V | 47249715 - 47265234 | [(James and Davey, 2009; Raza et al., 2016; van Wyk and Malan, 1988)](https://paperpile.com/c/DAmyjC/nHs1+nETP+tb9y) |
| pgp-2 / pgp-a ^2^ | [HCOI00025600](https://parasite.wormbase.org/Haemonchus_contortus_prjeb506/Gene/Summary?g=HCOI00025600) | I | 5849505 - 5868032 | [(Raza et al., 2016)](https://paperpile.com/c/DAmyjC/nETP) |
| pgp-11 | [HCOI00622400](https://parasite.wormbase.org/Haemonchus_contortus_prjeb506/Gene/Summary?g=HCOI00622400) | II | 31321675 - 31344154 | [(Raza et al., 2016)](https://paperpile.com/c/DAmyjC/nETP) |
| unc-38 | [HCOI01939400](https://parasite.wormbase.org/Haemonchus_contortus_prjeb506/Gene/Summary?g=HCOI01939400) | I | 25118411 - 25125593 |  |
| unc-7 ^1^ | [HPLM_0000412001](https://parasite.wormbase.org/Haemonchus_placei_prjeb509/Gene/Summary?g=HPLM_0000412001) |  |  | [(Dent et al., 2000)](https://paperpile.com/c/DAmyjC/Ip09) |
| unc-9 ^1^ | HCOI02075100 | Xb | 4410309 - 4424525 | [(Dent et al., 2000)](https://paperpile.com/c/DAmyjC/Ip09) |
| che-11 | HCOI00062600 | II | 3237676 - 3245201 |  |
| osm-6 | HCOI00937900 | V | 28370676 - 28397433 |  |
| pgp-3 | [HCOI00117000](https://parasite.wormbase.org/Haemonchus_contortus_prjeb506/Gene/Summary?g=HCOI00117000) | II | 11888372 - 11913805 | [(Raza et al., 2016)](https://paperpile.com/c/DAmyjC/nETP) |
| haf-6 | [HCOI01007100](https://parasite.wormbase.org/Haemonchus_contortus_prjeb506/Gene/Summary?g=HCOI01007100) | IV | 44336050 - 44341372 | [(Raza et al., 2016)](https://paperpile.com/c/DAmyjC/nETP) |
| pgp-13 / pgp-12 ^1^ | [HCOI01115500](https://parasite.wormbase.org/Haemonchus_contortus_prjeb506/Gene/Summary?g=HCOI01115500) | II | 10272077 - 10286519 | [(David et al., 2018)](https://paperpile.com/c/DAmyjC/vj6u) |
| mrp-1 ^1^ | [HCOI01904000](https://parasite.wormbase.org/Haemonchus_contortus_prjeb506/Gene/Summary?g=HCOI01904000) | Xb | 3325452 - 3334565 | [(James and Davey, 2009)](https://paperpile.com/c/DAmyjC/tb9y) |

1. *C. elegans* genes
2. Alternate names in the literature
3. Gene IDs correspond to the annotated gene in the published version of the MHco3(ISE) genome [(Laing et al., 2013)](https://paperpile.com/c/DAmyjC/wMz6).
4. Coordinates are approximate, as they represent the transfer of coordinates from the published version based on homology, and therefore may have changed depending on the conservation of the gene structure in the genome between the published and updated genome version used here.
